# Correction: Aghaei et al. The Role of BiP and the IRE1α-XBP1 Axis in Rhabdomyosarcoma Pathology. *Cancers* 2021, *13*, 4927

**DOI:** 10.3390/cancers17111883

**Published:** 2025-06-04

**Authors:** Mahmoud Aghaei, Ahmad Nasimian, Marveh Rahmati, Philip Kawalec, Filip Machaj, Jakub Rosik, Bhavya Bhushan, S. Zahra Bathaie, Negar Azarpira, Marek J. Łos, Afshin Samali, David Perrin, Joseph W. Gordon, Saeid Ghavami

**Affiliations:** 1 Department of Clinical Biochemistry, School of Pharmacy and Pharmaceutical Sciences, Isfahan University of Medical Sciences, Isfahan 81746-73461, Iran; parsa16657@yahoo.com; 2 Department of Human Anatomy and Cell Science, University of Manitoba College of Medicine, Winnipeg, MB R3E 0V9, Canada; nnasimiann@gmail.com (A.N.); kawalecp@myumanitoba.ca (P.K.); machajf@edu.pum.edu.pl (F.M.); jakubrosik@edu.pum.edu.pl (J.R.); bhavya.bhushan@mail.mcgill.ca (B.B.); 3 Department of Clinical Biochemistry, Faculty of Medical Sciences, Tarbiat Modares University, Tehran 14155-331, Iran; bathai_z@modares.ac.ir; 4 Cancer Biology Research Center, Tehran University of Medical Sciences, Tehran 14197-33141, Iran; m_rahmati@tums.ac.ir; 5 Institute for Natural Products and Medicinal Plants, Tarbiat Modares University, Tehran 14155-331, Iran; 6 Transplant Research Center, Shiraz University of Medical Sciences, Shiraz 7134814336, Iran; negarazarpira@gmail.com; 7 Biotechnology Center, Silesian University of Technology, 44-100 Gliwice, Poland; mjelos@gmail.com; 8 Apoptosis Research Centre, School of Natural Sciences, National University of Ireland, H91 W2TY Galway, Ireland; afshin.samali@nuigalway.ie; 9 Section of Orthopaedic Surgery, Department of Surgery, University of Manitoba, Winnipeg, MB R3E 0V9, Canada; david.perrin@umanitoba.ca; 10 College of Nursing, Rady Faculty of Health Science, University of Manitoba, Winnipeg, MB R3E 0V9, Canada; joseph.gordon@umanitoba.ca; 11 Research Institutes of Oncology and Hematology, Cancer Care Manitoba-University of Manitoba, Winnipeg, MB R3E 0V9, Canada; 12 Biology of Breathing Theme, Children Hospital Research Institute of Manitoba, University of Manitoba, Winnipeg, MB R3E 0V9, Canada; 13 Autophagy Research Center, Shiraz University of Medical Sciences, Shiraz 7134845794, Iran; 14 Faculty of Medicine, Katowice School of Technology, 40-555 Katowice, Poland

## Author's Information Correction

There was an error in the original publication [1]. Author name “Marek J. Los” should be corrected to “Marek J. Łos”. The post code in aff 7 should be corrected to 44-100.

## Text Correction

One of the antibodies information reported in “Section 2.2 Immunohistochemistry” was incorrect. Specifically, the catalog number for the anti-XBP1 antibody was incorrectly listed as ab96481 (Abcam). The correct antibody used in all experiments to detect XBP1 and sXBP1 was ab37152 (Abcam), a rabbit polyclonal antibody that detects both spliced (sXBP1, nuclear) and unspliced (XBP1, cytosolic) isoforms of XBP1. This correction does not alter the interpretation of the data but is important to ensure the transparency and reproducibility of the methods described.

A correction has been made to Section 2.2 Immunohistochemistry:

“TMA slides (5 μm thick) were deparaffinized in 60 °C for 30 min and subsequently rehydrated in xylene with a gradient alcohol series. Heat-mediated antigen retrieval (0.01 M sodium citrate buffer, pH 6.0) was performed as described previously [22,26,27]. In order to eliminate background interference, the slides were washed with phosphate-buffered saline (PBS) and blocked with blocking solution (1.5 mL Maleic Acid Buffer, 0.5 mL FBS, 0.5 mL stock blocking solution, 50 μL 10% Tween-20, and 2.5 mL PBS) at room temperature for 30 min. After washing with PBS, the slides were incubated in freshly prepared 3% H_2_O_2_ to eliminate endogenous hydrogen peroxidases. The TMA slides were incubated with Avidin blocking solution (Vector SP-2001, 15 min) and then with Biotin blocking solution (Vector WP-2001, 15 min). Next, the slides were incubated overnight (4 °C) with mouse mAb against IRE1 (1:100; cat. no. ab96481; Abcam), rabbit mAb against BiP (1:200; cat. no. #3177, Cell Signaling; Danvers, MA USA), and rabbit polyclonal antibody against cytosolic and nuclear XBP1 (1:200; cat. no. ab37152; Abcam). Following thorough washing with PBS, the slides were incubated with biotinylated secondary antibody (corresponding to the primary antibody) for 1 h at room temperature, washed with PBS, and incubated with horseradish peroxidase-labeled streptavidin (1:200) for 30 min at room temperature. Finally, the slides were incubated with a 3,3′-diaminobenzidine-peroxidase substrate for 2 min at room temperature and counterstained with Mayer Hematoxylin (Vector H-3404; 10 drops in 1.25 mL PBS) for 1–4 min. In order to exclude any nonspecific staining of the secondary antibodies, negative controls were performed without the addition of any primary antibody.”

## Updated Data Availability Statement

A correction has been made to Data Availability Statement:

**Data Availability Statement:** The data supporting the findings of this study are available in the Supplementary Material (Table S1). To ensure transparency and allow readers to directly review the original immunohistochemistry data, all raw images, including those presented in Figures 1–4, have been deposited in the Dryad digital repository. These can be accessed via the following DOI: https://datadryad.org/dataset/doi:10.5061/dryad.d2547d8c6.

The authors state that the scientific conclusions are unaffected. This correction was approved by the Academic Editor. The original publication has also been updated.
